# In vitro activities of thiazolidione derivatives combined with daptomycin against clinical *Enterococcus faecium* strains

**DOI:** 10.1186/s12866-021-02423-8

**Published:** 2022-01-07

**Authors:** Zhong Chen, Yanpeng Xiong, Yuanyuan Tang, Yuxi Zhao, Junwen Chen, Jinxin Zheng, Yang Wu, Qiwen Deng, Di Qu, Zhijian Yu

**Affiliations:** 1 Department of Infectious Diseases and the Key Lab of Endogenous Infection, Shenzhen Nanshan People’s Hospital and The 6th Affiliated Hospital of Shenzhen University Health Science Center, Shenzhen, 518052 China; 2 Key Laboratory of Medical Molecular Virology of Ministries of Education and Health, Department of Medical Microbiology and Parasitology, School of Basic Medical Science and Institutes of Biomedical Sciences, Shanghai Medical College of Fudan University, Shanghai, 200032 China

**Keywords:** *Enterococcus faecium*, Antibacterial, Anti-biofilm, Biofilm formation

## Abstract

**Background:**

Previous reports have demonstrated two thiazolidione derivatives (H2-60 and H2-81) can robustly inhibit the planktonic growth and biofilm formation of *S. epidermidis* and *S. aureus* by targeting the histidine kinase YycG**.** Whereas the antibacterial and anti-biofilm activity of these two thiazolidione derivatives (H2-60 and H2-81) against *Enterococcus faecium* remains elusive. Here, the pET28a-YycG recombinant plasmid were in vitro expressed in *E. coli* competent cell BL21 (DE3) and induced to express YycG’ protein (conding HisKA and HATPase_c domain) by 0.5 mM IPTG and was purified by Ni – NTA agarose and then for the autophosphorylation test. Antimicrobial testing and time-killing assay were also be determined. Anti-biofilm activity of two derivatives with sub-MIC concentration towards positive biofilm producers of clinical *E. faecium* were detected using polystyrene microtiter plate and CLSM.

**Results:**

The MICs of H2-60 and H2-81 in the clinical isolates of *E. faecium* were in the range from 3.125 mg/L to 25 mg/L. Moreover, either H2-60 or H2-81 showed the excellent bactericidal activity against *E. faecium* with monotherapy or its combination with daptomycin by time-killing assay. *E. faecium* planktonic cells can be decreased by H2-60 or H2-81 for more than 3 × log10 CFU/mL after 24 h treatment when combined with daptomycin. Furthermore, over 90% of *E. faecium* biofilm formation could markedly be inhibited by H2-60 and H2-81 at 1/4 × MIC value. In addition, the frequency of the eradicated viable cells embedded in mature biofilm were evaluated by the confocal laser microscopy, suggesting that of H2-60 combined with ampicillin or daptomycin was significantly high when compared with single treatment (78.17 and 74.48% vs. 41.59%, respectively, *P* < 0.01).

**Conclusion:**

These two thiazolidione derivatives (H2-60 and H2-81) could directly impact the kinase phosphoration activity of YycG of *E. faecium*. H2-60 combined with daptomycin exhibit the excellent antibacterial and anti-biofilm activity against *E. faecium* by targeting YycG.

**Supplementary Information:**

The online version contains supplementary material available at 10.1186/s12866-021-02423-8.

## Background

*Enterococcus* spp. is known to be capable of causing nosocomial and life-threatening infections in human. *Enterococcus faecium* infection can result in a variety of infectious diseases, including intra-abdominal and pelvic regions infections, urinary tract infections, implantable infections, biofilm-related infections, etc. [1]. Pathogens causing hospital acquired infections in the US during 2011–2014 showed an overall contribution of *E. faecium* of 3.7% [2]. A large proportion (39.9%) of enterococcal bacteremia was caused by *E. faecium* [3]*.* Multidrug resistance has been increased globally that is considered public health threat. Several recent studies revealed the emergence of multidrug-resistant bacterial pathogens from different origins including humans, poultry, cattle, and fish that increases the need for new antimicrobial alternatives to the commonly used old antimicrobial agents [4–6]. Recently, the increasing reports of multidrug resistant *E. faecium* strains, including vancomycin-,daptomycin- or linezolid-resistant strains, has attracted the attention of the scientific community [7, 8]. Previous researchers have reported that virulence factors played important roles in the pathogenicity of enterococci [9]. These virulence factors could help *E. faecium* to break the host immune defense system to accelerate the bacteria to colonize on the infection site and thereby leading to cause *E. faecium* infection [10]. Virulence factors also have been proved to enhance the biofilm formation of bacteria and cause biofim-related endocarditis and urinary tract infections [11–13].

The ideal antimicrobial agent for infection treatments may possess the following characteristics: (1) Highly selectivity for bacteria with clear and conserved target. (2) Low toxicity in vivo. (3) Not easily resistant to bacteria. (4) Potent antimicrobial activity or even broad-spectrum effects.

In addition to focusing on increasing drug resistance, *E. faecium* can also form biofilms, which could often enhance the difficulty of antimicrobial treatment to suppress the protracted and chronic infections [14–17]. The biofilm formation limits the diffusion and penetration of bacteria due to the aggregation of extracellular polymeric substances (EPS) matrix. One of the most difficulties for the treatment of biofilm-related infection is to eradicate established biofilms on the surfaces of devices or cavities [18]. Therefore, the development of novel antimicrobial agents against the planktonic growth and biofilm formation of *E. faecium* would be beneficial for clinicians to improve the prognosis of these infections.

Previous studies have confirmed the decisive role of two-component systems (TCSs) of bacteria in the planktonic growth and biofilm formation of a variety of bacteria [19, 20]. Therefore, TCSs have aroused the increasing attention as potential targets for the development of antibacterial and anti-biofilm drugs. YycFG is one of the TCSs involved in biofilm formation, cell wall metabolism, resistance and regulation of virulence factors in *Bacillus subtilis*, *Staphylococcus aureus*, *Staphylococcus epidermidis* and *Streptococcus* [21–24]. The histidine kinase YycG is a transmembrane protein and the kinase ATP-binding domain (HATPase_c domain) can sense external signals and is activated by extracellular signals, which subsequently resulting in the phosphorylation of the histidine site (HisKA domain) of YycG. By targeting YycG, small molecule compounds with antibacterial activity have been effectively developed against the gram-positive bacteria such as *B. subtilis*, *S. aureus* [25, 26], *S. epidermidis* and *Streptococcus* [8, 27]. Therefore, YycG may be a potential alternative target for the development of antimicrobial agents. Our group previously described the bactericidal and anti-biofilm activities of two YycG inhibitors, H2-60 and H2-81, by targeting the HATPase_c domain of *S. epidermidis* YycG [28], *S. aureus* [29]. But it is still unclear whether the two thiazolidione derivatives (H2-60 and H2-81) are capable to block the planktonic growth and biofilm formation in *E. faecium*.

The aim of this study was to investigate the phosphorylation inhibitory activities of the thiazolidione derivatives (H2-60 and H2-81) to the YycG protein with kinase ATP-binding site (HATPase_c domain). The antibacterial and anti-biofilm activities of these two derivatives against *E. faecium* have been evaluated. More important, the potential application prospects of the combination therapy of H2-60 and H2-81 with the common antibiotics on biofilm formation of *E. faecium* were also assessed.

## Results

### The inhibitory effect of two thiazolidione derivatives on the kinase activity of the recombinant *E. faecium* YycG’

**Fig. 1:**
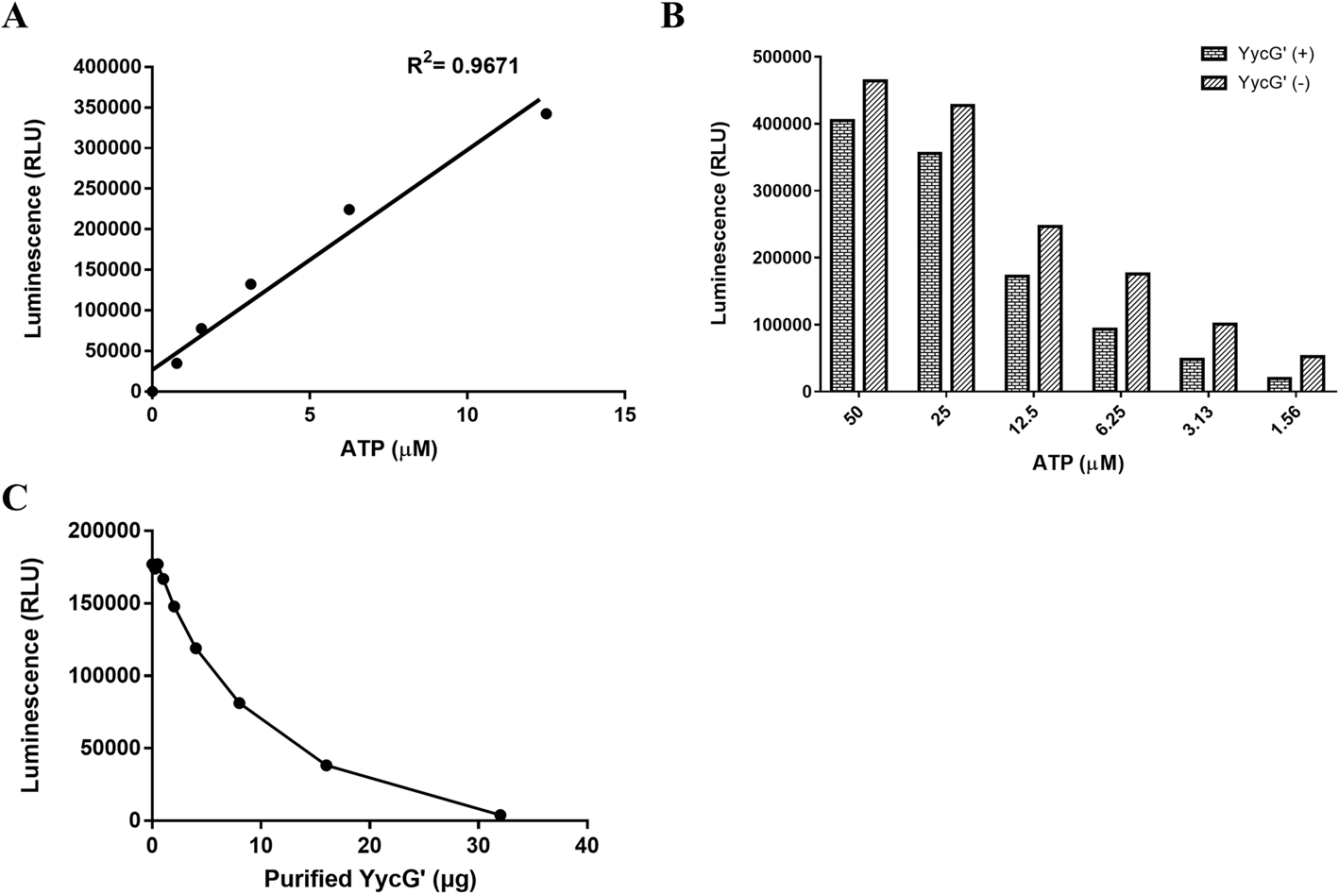
Validation of the correlation ship between YycG’ recombinant protein kinase and ATP consumption in vitro*.* Luminescence had a direct correlation with the consumption of ATP. **A** Data showed that a positive relationship between the RLU and the consumption of ATP with variant concentrations from 0 to 12.5 μM measured with the Kinase-Glo® Assay (*R*^2^ = 0.9671). **B** 3 μg of YycG’ recombinant protein was added into reaction system containing variant ATP concentrations (1.56 ~ 50 μM), systems without YycG’ present were used as control. **C** Variant amount of YycG’ proteins (0.5 ~ 32 μg) were added into the systems with 4 μM ATP. Data (means ± SDs) were performed in triplicate independently

In order to verify the impact of two thiazolidione derivatives on the kinase activity of the recombinant YycG’ proteins of *E. faeciumin* in vitro, Kinase-Glo® Luminescent Kinase (Promega) was adopted to detect the correlation between relative light unit (RLU) and ATP content in the reaction system. Our data indicated the close correlation between the ATP concentration and the RLU (*R*^2^ = 0.9671, Fig. 1A). Moreover, the kinase activity of the recombinant protein YycG’ were also verified in vitro, suggesting the optimized amount of ATP (shown in Fig. 1B) and 3 μg YycG’ protein were suitable for the addition with the various concentration gradients of ATP (1.56–50 μM) for the further detection of the kinase activity. RLU value decreased significantly in comparison to the control group without the addition of YycG’ protein in addition of ATP concentration between 3.13–6.25 μM (Fig. 1B). RLU value was also significantly decreased with the increasing concentrations of YycG’ proteins (Fig. 1C). Based on the above results, the effect of two thiazolidione derivatives on the kinase activity of YycG were detected with the concentration of 4 μM ATP and 3 μg recombinant protein, suggesting the concentration-dependent inhibition of the activity of YycG’ protein kinase by H2-60 and H2-81 respectively. The IC_50_ values (the concentration of compound inhibiting 50% of YycG’ kinase activity) of H2-60 and H2-81 respectively were 25.4 and 26.2 μM respectively (Table 1).

**Table 1:** Biological activities of the two thiazolidione derivatives

Compounds	Molecular formula	MW	MIC^**a**^ (μM)	MBC^**a**^ (μM)	IC_**50**_^**b**^ (μM)
H2-60	C_27_H_16_F_2_N_2_O_3_S_2_	518	3.13	12.5	25.4
H2-81	C_27_H_17_FN_2_O_3_S_2_	500	3.13	12.5	26.2
Ampicillin	C_16_H_18_N_3_NaO_4_S	371	2.70	10.8	ND
Vancomycin	C_66_H_75_Cl_2_N_9_O_24_·HCl	1486	0.67	2.7	ND
Linezolid	C_16_H_20_FN_3_O_4_	337	5.93	23.7	ND
Daptomycin	C_72_H_101_N_17_O_26_	1621	0.62	2.48	ND

*MW* molecular weight, *ND* not detect

^a^ MIC (minimal inhibitory concentration) and MBC (minimal bactericidal concentration) against *E. faecium* EF16M64

^b^IC_50_ represents the concentration of the YycG in inhibitors that inhibited 50% of the autophosphorylation of YycG′ detected by Kinase-Glo Luminescent kit

### Antibacterial activity of two thiazolidione derivatives (H2-60 and H2-81) against *E. faecium*

**Fig. 2:**
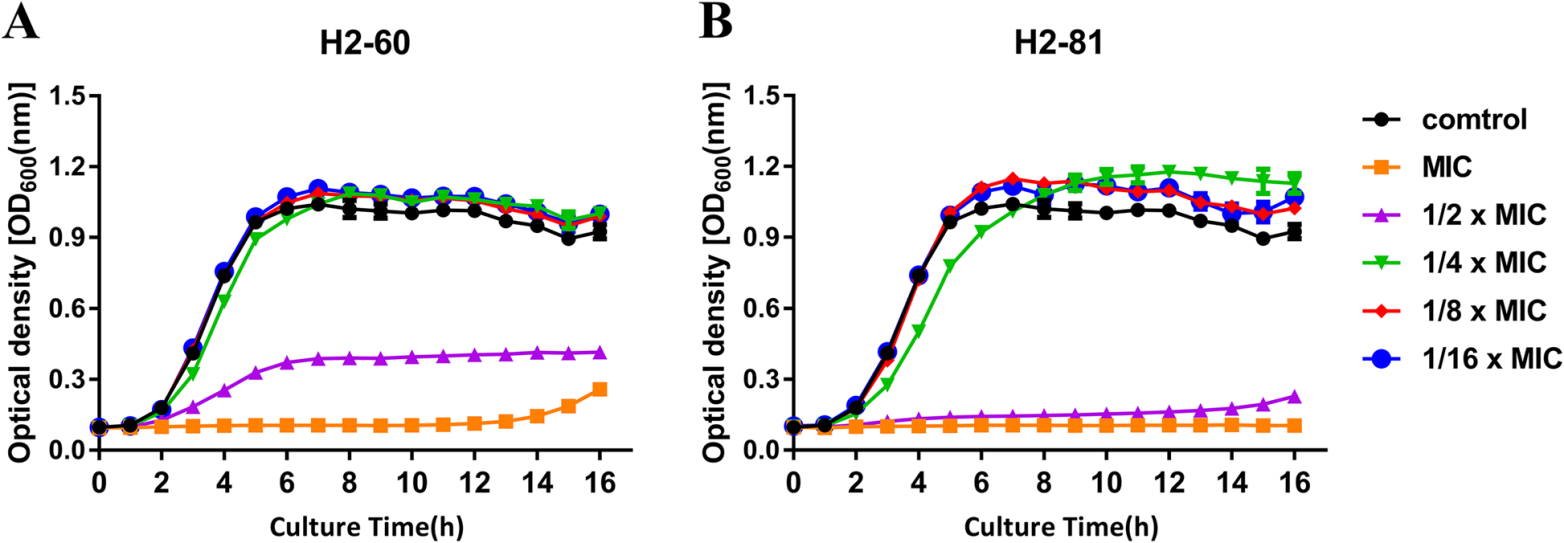
Growth curves of thiazolidione derivatives against planktonic bacteria of *E. faecium* EF16M64 in sub-MIC concentrations. *The E. faecim* EF16M64 was treated with **a** H2-60 and **b** H2-81 at 1×, 1/2×, 1/4×, 1/8×, and 1/16× MICs for 24 h. The growth of their planktonic cells was determined by optical density at 600 nm (OD_600_). Data (means ± SDs) were performed in triplicate independently

The MIC values of H2-60 and H2-81 determined by broth microdilution method against 102 non-repetitive clinical *E. faecium* strains were ranging from 3.13 μM to 25 μM*.* Moreover, the MIC_50_/MIC_90_ values of these clinical *E. faecium* strains were 6.25/6.25 μM for H2-60 and 6.25/12.5 μM for H2-81 respectively (Table 2). The MIC and the MBC value for *E. faecium* EF16M64 was 3.13 μM and12.5 μM respectively. The MIC and the MBC values of ampicillin, vancomycin, linezolid and daptomycin against *E. faecium* EF16M64 were listed in Supplementary Table S1. Another clinical antimicrobial agents against 102 *E. feacium* strains were also determined, showing the MDR phenotypes in Supplementary Table S3, we can see H2-60 and H2-81 also had good activities against multidrug-resistant strains, including vancomycin or linezolid non-susceptible strains. The effect of H2-60 and H2-81 on planktonic bacterial growth at different sub-inhibitory concentrations were evaluated, suggesting the unaffected growth of planktonic bacteria by these two derivatives at the concentration of 1/4 × MIC or below (Table 1 and Fig.2).

**Table 2:** MIC values of thiazolidione derivatives against 102 clinical *E. faecium* strains

Compounds	No of the MIC values creep				MIC_**50**_ (μM)	MIC_**90**_ (μM)
	3.13	6.25	12.5	25		
H2-60	2	79	17	4	6.25	6.25
H2-81	2	48	47	5	12.5	12.5

### Time-killing assay of two thiazolidione derivatives (H2-60 and H2-81) against *E. faecium*

**Fig. 3:**
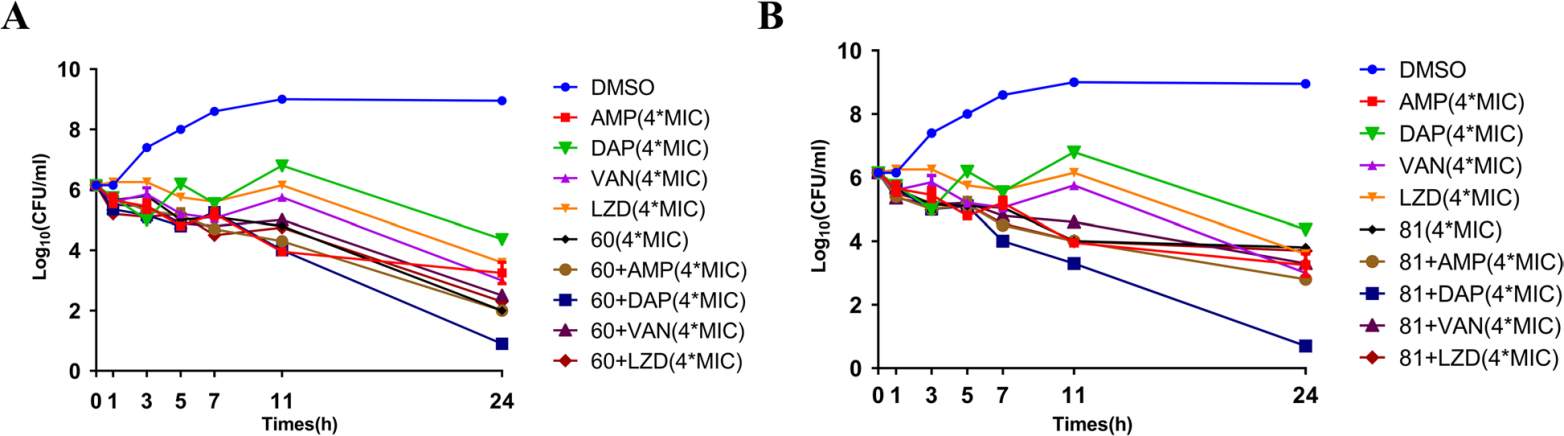
Time-killing curve of thiazolidione derivatives combined with ampicillin, vancomycin, linezolid, or daptomycin against *E. faecium* EF16M64. Antibacterial activities of **a** H2-60 and **b** H2-81 on *E. faecim* EF16M64 planktonic cells were determined by time-killing study. The data presented was the average of three independent repeats (mean ± SD). All chemicals or antimicrobials were used at 4 × MIC. AMP ampicillin, DAP daptomycin, VAN vancomycin, LZD linezolid

Time**-**killing assay were performed with two thiazolidione derivatives (H2-60 and H2-81) alone or combined with ampicillin, vancomycin, linezolid, or daptomycin at concentrations of 4 × MIC respectively. The original amount of viable bacteria cells of all groups were 6.1 log_10_CFU/mL counts. We observed the bactericidal effect of H2-60 and H2-81 was equivalent to that of ampicillin, vancomycin and linezolid within 24 h, with a reduction of more than 2 orders of magnitudes from baseline of 6.1 to 2.0–3.8 log_10_CFU/mL. Worthy of our attention, when H2-60 or H2-81 combined with daptomycin, the amount of viable bacteria cells could be decreased from baseline of 6.1 to 3.3–4.0 log_10_CFU/mL within 11 h, and even to 0.7–0.9 log_10_CFU/mL within 24 h (Fig. 3).

### Anti-biofilm activity of two thiazolidione derivatives (H2-60 and H2-81) against *E. faecium*

The biofilm formation of 102 *E. faecium* strains from different sources was assayed using polystyrene microtiter plate. EF16M2 strain (OD_570_ ≈ 0.207 ± 0.03) was regarded as negative control and OD_570_ cut-off (OD_c_) value was calculated to be 0.297. 52.9% (54/102) of the clinical *E. faecium* strains was shown positively with biofilm formation, 7.8% (8/102) were shown with strong positive biofilm phenotype, 11.8% (12/102) were medium positive biofilm phenotype, and 33.3% (34/102) could form the weak positive biofilm (Table 3).

**Table 3:** Occurrence of *E. faecium* biofilm formation by clinical source

Clinical source (no. isolates tested)	No. (%) of isolates with biofilm phenotype				
	Strong	medium	Strong or medium	Weak	All positive
Blood (9)	1 (11.1)	1 (11.1)	2 (22.2)	3 (33.3)	5 (55.6)
Urine (55)	3 (5.5)	7 (12.7)	10 (18.2)	19 (34.5)	29 (52.7)
Pus or secretions (10)	2 (20.0)	0 (0.0)	2 (20.0)	3 (30.0)	5 (50.0)
Bile (6)	1 (16.7)	1 (16.7)	2 (33.3)	2 (33.3)	4 (66.7)
Other^a^ (22)	1 (4.5)	3 (13.6)	4 (18.2)	7 (31.8)	11 (50.0)
Total (102)	8 (7.8)	12 (11.8)	20 (19.6)	34 (33.3)	54 (52.9)

^a^ Other sources contained sputum, ear swab, feces, catheter, pleural effusion, ascites fluid, drainage fluid and bronchoalveolar lavage fluid

**Fig. 4:**
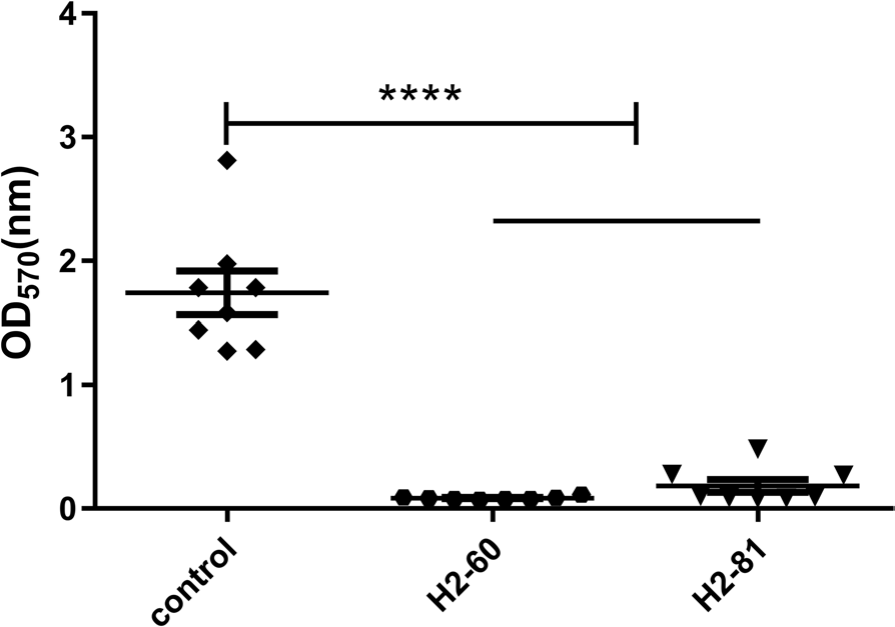
Two thiazolidione derivatives inhibited the biofilm formation of *E. faecium* strains in sub-MIC concentration. *E. faecium* strains (8 strains, strong biofilm producers) were treated with H2-60 and H2-81 at 1/4 × MIC for 24 h, and the biofilm biomasses were determined by crystal violet staining. The experiments were repeated in triplicate, each data represented as the average of the results of three independent experiments. ****: vs. control group, *P* < 0.0001. (nonparametric Mann–Whitney U test)

The growth curves demonstrated that H2-60 or H2-81 did not affect the growth of *E. faecium* EF16M64 planktonic cells at 1/4 × MIC or below (shown in Fig. 2). Eight strains of *E. faecium* with the range of positive biofilm formation OD_570_ from 1.27 to 2.814 were selected to evaluate the inhibitory effect of H2-60 and H2-81at sub-MIC concentration (1/4 × MIC), indicating that the significantly reduced biofilm formation when treated with 1/4 × MIC of H2-60 or H2-81 for 24 h respectively (Fig. 4, *P* < 0.0001, Student’s t test) .

### Eradication of mature biofilm of *E. faecium* by two thiazolidione derivatives (H2-60 and H2-81) using CLSM

**Fig. 5:**
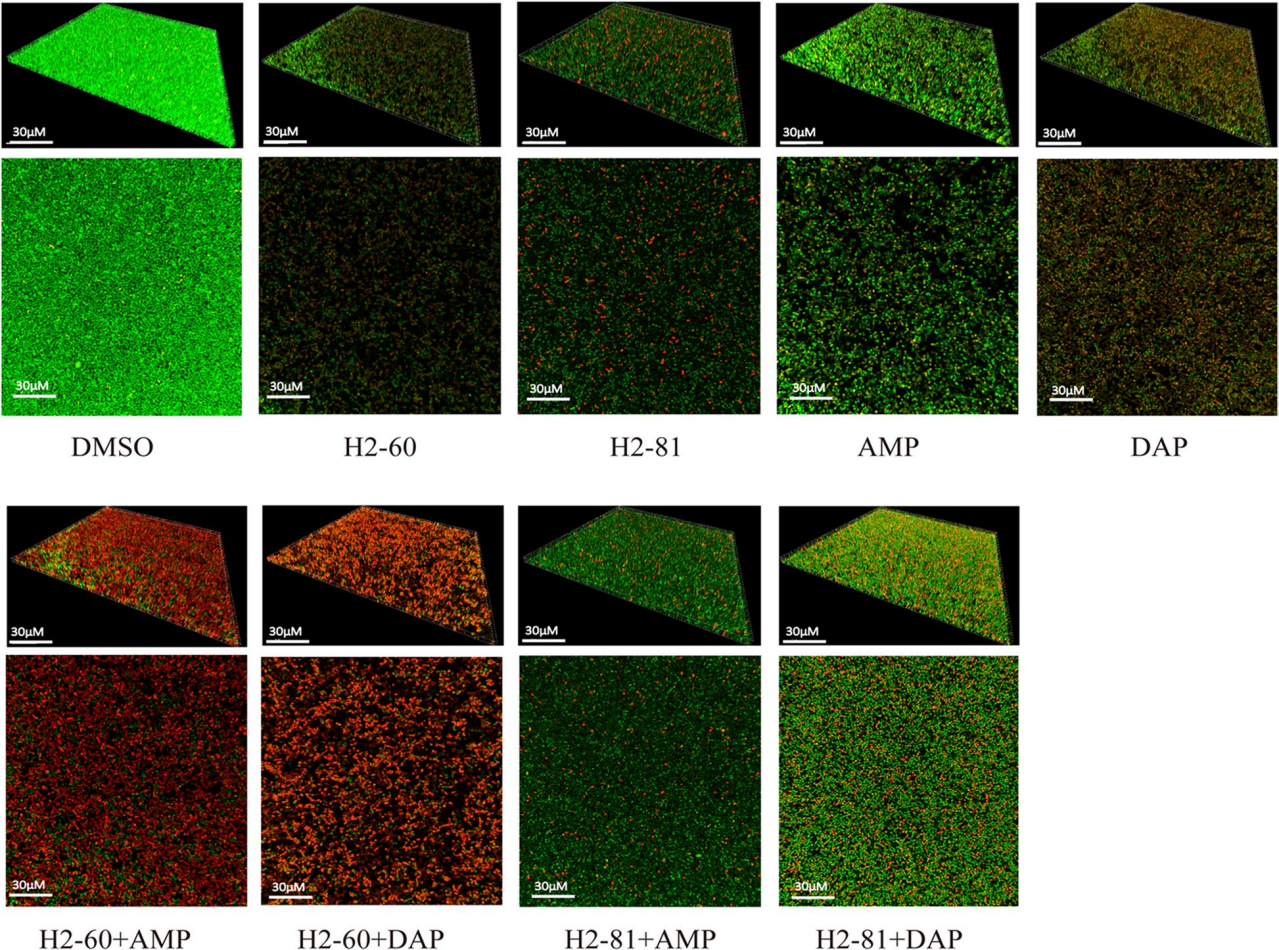
Effects of the two thiazolidione derivatives on mature biofilm of *E. faecium* EF16M64. The established biofilms of *E. faecium* EF16M64 stained with SYTO9 and propidium iodide (PI) were observed under a confocal laser scanning microscope (CLSM, Leica). Images were analyzed by IMARIS 7.0.0 software (Bitplane). Cells stained with green fluorescence (SYTO9) were viable and with red fluorescence (PI) were dead. Experiments were performed in three independent repeats and representative images were shown. DAP: daptomycin; AMP: ampicillin]

The optimal concentration of the inhibitor (1 × MIC) and common antibiotics (8 × MIC) was finally defined by exploring the ablating effect of inhibitors on mature biofilm of *E. faecium* at different concentration gradients (1/4 ×, 1/2 ×, 1 ×, 2 ×, 4 ×, 8 ×, 16 ×, 32 × MICs). Thus, the 1 × MIC of H2-60 or H2-81 was used to explore their effect on the mature biofilm of *E. faecium*, and compared with ampicillin or daptomycin (8 × MIC). CLSM were performed for the quantification of the amount of the viable bacteria cells, suggesting the amount of viable biofilm-embedded bacteria cells in the mature biofilm were reduced to 41.6 and 29.0% by H2-60 and H2-81 respectively. Interestingly, 78.17 and 74.48% of viable biofilm-embedded bacteria cells in mature biofilms could be significantly eradicated by H2-60 combined with ampicillin or daptomycin in comparison to H2-60 alone (Fig. 5 and Table 4).

**Table 4:** The proportion of viable cells or dead cells within mature biofilm using ImageJ software

Compounds	SYTO9	PI	PI/Total
H2-60	19.785 ± 25.843	12.374 ± 18.152	41.59%
H2-81	24.691 ± 25.063	10.065 ± 25.322	28.96%
AMP	41.525 ± 49.978	13.659 ± 26.067	24.75%
DAP	28.258 ± 28.01	27.802 ± 30.489	49.6%
H2-60 + AMP	13.905 ± 22.923	49.795 ± 53.822	78.17%^a^
H2-60 + DAP	18.878 ± 25.598	55.093 ± 64.402	74.48%^b^
H2-81 + AMP	37.991 ± 29.629	8.66 ± 20.861	18.56%
H2-81 + DAP	67.603 ± 55.643	36.008 ± 51.793	34.75%
DMSO	131.508 ± 57.004	2.565 ± 10.567	2.0%

^a^ H2-60 + AMP group vs. H2-60 group, *P <* 0.01; ^b^ H2-60 + DAP group vs. H2-60 group, *P <* 0.01 (Student’s *t* test)

## Discussions

The rapid emergence of multidrug-resistant *Enterococcus* spp. has limited the available drug options for the treatment of *Enterococcus* infections in human. Currently, just few antimicrobial agents are very effective for the treatment of biofilm-associated infections such as endocarditis, intravascular catheter-related infections, etc. Therefore, it’s urgent for us to develop the effective compounds for the improvement of the clinical outcome of the biofilm-associated infections caused by *Enterococcus* spp.

YycFG TCS has been shown to play a key role in planktonic growth and biofilm formation in a variety of Gram-positive bacteria [21–23]. Previous reports have indicated the excellent antibacterial and anti-biofilm activities of H2-60 and H2-81against *S. epidermidis*, *S. aureus* by targeting the histidine kinase YycG of *S. epidermidis* with low cytotoxicity [22, 29]. Bioinformatics analysis data showed the high homologous characteristics of the YycG-encoded protein of *E. faecium* compared with that in *S. epidermidis* and *S. aureus* (about 60%, Supplementary Table S2). We hypothesize that two thiazolidione derivatives targeting YycG can also be used for *E. faecium* infection. In this study, our data showed the low MIC values of H2-60 and H2-81 against clinical *E. faecium* isolates (both MIC_50_/MIC_90_: 6.25/12.5 μM). Time-killing assay further indicated the bactericidal activity of H2-60 and H2-81 equivalent to that with ampicillin, vancomycin, and linezolid. IC_50_ results supported the binding affinity of H2-60 and H2-81 with the recombinant YycG’ protein of *E. faecium*, which maybe the same target sites in *S. epidermidis* of YycG.

The present research also found that H2-60 and H2-81 were also effective against different MDR strains, including vancomycin or linezolid non-susceptible strains. Daptomycin and linezolid were first-line therapeutic option for vancomycin-resistant *E. faecium* infection and high dose are often recommended [30], but more evidences have been demonstrated that daptomycin use was not associated with better microbiological cure than linezolid and have high mortality [31]. Meanwhile, Time-killing curve of daptomycin treatment alone also showed no bactericidal activity [32], which were consistent with our results. Interestingly, time-killing results further indicated both H2-60 and H2-81 combined with daptomycin could had strong bactericidal effect and sustained for over 24 h, suggesting the possible synergetic role of these two thiazolidione derivatives with daptomycin. Daptomycin is a lipopeptide antibiotic used for gram-positive bacterial infection, which can block the peptidoglycan biosynthesis of the bacterial cell wall by disturbing the transport of amino acids of the cell membrane. Multiple reports have demonstrated the involvement of YycG in the degradation of *S. aureus* cell wall by hydrolyzing peptidoglycan, and nucleotide insertion mutation in the *yycG* gene participate in the reduced daptomycin susceptibility in *S. aureus*. Therefore, we speculated that H2-61 and H2-80 can exert a synergistic bactericidal role with daptomycin by interfering with the synthesis of bacterial cell wall, but their possible synergetic mechanism needs to be further elucidated.

Biofilm matrix is often composed of polysaccharides, extracellular DNA (EDNA), a variety of lipids and proteins et al. It’s difficult to kill the viable bacteria cells that embedded in biofilm from the clinical environment. Currently, limited effective antibiotic can be used for the treatment of biofilm associated infections and the eradication of mature biofilms still remains an urgent clinical problem. This study observed that H2-60 and H2-81 had significant inhibitory effect on the *E. faecium* biofilm formation and the viable bacteria embedded in the established biofilm detected by CLSM can be eradicated by the H2-60 monotherapy. It is also found for the first time that the combination of H2-60 with daptomycin could enhance the clearance capacity of the viable bacteria embedded in the mature biofilms. This may be that daptomycin disrupted cell membrane stability of *E. faecium* synergistically with H2-60 targeting YycG to interfere with biofilm formation, which is facilitate to the entry of daptomycin and H2-60 into the established biofilm to kill more viable bacteria.

We also observed the phenomenon that the H2-81 had worse killing effects on adherent cells in the established biofilm than that of H2-60. Which showed the opposite effect as the derivatives agnist *S. epidermidi* [28] and *S. aureus* [29]*.* This study also indicated that the anti-biofilm activities of ampicillin and daptomycin had been attenuated with H2-81 condition but been enhanced in that of H2-60. Our previous study had found that halogen substituents (F or Cl) on phenyl rings of thiazolidine core structure could improve antibacterial or anti-biofilm activities than its leading compounds [28]. In terms of molecular structure, H2-81 lacked an F element on its phenyl rings, which may change its drug structure-activity and molecular symmetry, thus attenuating the anti-biofilm effect of ampicillin and daptomycin. Another possible reason maybe that ampicillin and itself had a strong bactericidal effect on *E. faecium*, reducing the counts of bacteria to a very low number in a short time, so that we can not observe the synergistic bactericidal effect. Therefore, the above results suggest that thiazolidione derivatives H2-60 might be used as an adjunct to antimicrobials with potential applications for the treatment of biofilm-associated infections caused by *E. faecium,* especially when combined with daptomycin.

## Conclusion

Two thiazolidione derivatives (H2-60 and H2-81) targeting the HATPase_c domain of *S. epidermidis* YycG can also bind to the YycG of *E. faecium*. Time-killing results demonstrated the synergetic bactericidal activity of H2-60 and H2-81 with daptomycin toward the planktonic growth of *E. faecium*. The combination therapy of H2-60 with daptomycin could enhance the bactericidal activity toward the viable bacteria embedded in the established *E. faecium* biofilm. Therefore, H2-60 is suitable used as an adjuvant drug to enhance the anti-biofilm activities of other antibiotics. It also suggests that thiazolidione derivatives targeting YycG combined with daptomycin may become a new strategy for the treatment of *E. faecium* infection and biofilm-related infections.

## Methods

### Bacteria strains and materials

One hundred two non-redundant clinical isolates of *E. faecium* were obtained from Shenzhen Nanshan People’s Hospital and the 6th Affiliated Hospital of Shenzhen University Health Science Center (Table 3 and Supplementary Table S3). These clinical strains were first identified by the Phoenix 100 automated microbiology system (BD, USA) and then sub-cultured generations were re-identified with matrix-assisted laser desorption ionization time-of-flight mass spectrometry (MALDI-TOFMS, IVD MALDI Biotyper, Germany) [33]. Phoenix 100 automated microbiology system was used for indentification and antimicrobial susceptibility testing of *E. faecium* by broth microdilution method. By using MALDI-TOFMS technoloty, the unique proteins and peptides of bacteria were arranged according to the molecular weight to form a unique proteome finger map of characteristic pattern peaks for strains identification. The common-used antibiotics, including vancomycin (catalog no. V2002), linezolid (catalog no. PZ0014), ampicillin (catalog no. A9518) and daptomycin (catalog no. SBR 00014), were purchased from Sigma Aldrich (Shanghai, China). Ampicillin was the first choice for *E. faecium* infection, vancomycin, linezolid, and daptomycin were often used in multidrug resistant strains of *E. faecium* and other gram-positive bacterial infections. So we choose these four agents to compare their antimicrobial activities to two thiazolidione derivatives. Two YycG inhibitors of the thiazolidione derivatives (H2-60 and H2-81) were synthesized and obtained from WuXiAppTec Co., Ltd. (Shanghai, China). Mueller-Hinton Broth (MHB), CA-Mueller-Hinton Broth (CAMHB), Tryptone Soy Broth (TSB) and Agar were purchased from Oxoid Ltd. (Basingstoke, England). *E. faecalis* ATCC29212 (biofilm negative) were used as quality control for MIC determination and *E. faecium* EF16M64 were used as biofilm positive strains.

### Antimicrobial susceptibility testing and MBC assays

The *E. faecium* strains were cultured overnight on blood agar plates and these isolates were identified by the Phoenix 100 automated microbiology system (BD, Franklin Lakes, New Jersey, USA). The MIC values of two thiazolidione derivatives (H2-60 and H2-81), vancomycin, linezolid, ampicillin and daptomycin against *E. faecium* were determined by broth microdilution method according to the standards of Clinical and Laboratory Standards Institute (CLSI) in 2019. The susceptibilities of *E. faecalis* isolates to another clinical relevant antimicrobials (i.e., teicoplanin, tetracycline, erythromycin, nitrofurantoin, ciprofloxacin, high-level gentamicin and rifampicin) were determined by supporting gram-positive antimicrobial susceptibility test kit of BD automated microbiology system (No. PMIC-92). Briefly to describe the MICs detection of H2-60 and H2-81, two-fold serial dilutions of the compounds at final concentrations from 200 to 0.39 μM were prepared in 96-well microtiter plates (Falcon) containing 100 μL CAMHB medium. The medium was adjusted by sterile saline until the bacterial suspension density was equal to a 0.5 McFarland standard (∼1.0 × 10^8^ CFU/mL) and then diluted to 1:100 into CA-MHB medium. The diluted bacteria were added into 96 well plate (50 μL/well) and mixed and placed in 37 °C incubator and incubated at 220 rpm for 20–24 h. The interpretation of MIC was the lowest concentration without visible cells growth in the well. One hundred microliter of bacteria solution with or above MIC value was coated on a new MHA plate and incubated at 37 °C for 24 h for counting. The MBC value was represented as a 99.99% reduction of the original inoculum or the number of clones on each plate is ≤5. Besides, positive control (without compound) and negative control (without bacterial solution) were also set and *E. faecalis* ATCC29212 was used as quality control. MIC_50_/MIC_90_ was used to represent MIC value distribution of all the clinical strains. All experiments were performed in triplicate.

### Cloning, expression and purification of the YycG’ protein

The YycG’ fragment containing the cytoplasmic catalytic and ATP-binding domains (the HisKA and HATPase_c domain, see Fig. 1A) of YycG (377aa to 604aa) in *E. faecium* was amplified by PCR with the template of genomic DNA of *E. faecium* DO (Genbank No. NC_017960). The amplified primers were 5′-TTTGGATCCCGTCGTGAATTCGTCTCTAA − 3′ and 5′-TTTCTCGAGTAGGTTCATATGGCAGCGAGAT-3′ respectively. Subsequently, the fragment was digested with *BamHI* and *XhoI* (Thermo Fisher Scientific, Massachusetts, USA) and ligated into the corresponding sites of pET28 (a) to obtain pET28 (a)-YycG’. After being transformed into *E. coli* strain BL21 (DE3), this recombinant plasmid was induced to express YycG′ protein by 0.5 mM isopropyl-1-thio-β-D-galactopyranoside at 22 °C for 12 h. Then, the bacterial cells were disrupted by sonication and centrifuged, the supernatant was purified by Ni–NTA agarose (Qiagen, Los Angeles, CA, USA) using affinity chromatography method. The concentration of recombinant YycG’ protein was determined by the BCA Protein Assay Kit (Thermo Fisher Scientific, Massachusetts, USA), according to the manufacturer’s protocol.

### Inhibition assay for phosphorylation activity of the HATPase_c domain of YycG′

In order to verity interaction of two thiazolidione derivatives (H2-60 and H2-81) with the HATPase_c domain of YycG, impact of these two derivatives on the ATPase activity of the YycG′ protein were measured using the Kinase-Glo™ Luminescent Kinase Assay (Promega, Madison, USA). Briefly, 3 μg purified YycG′ protein was pre-incubated with a series of dilutions of the compounds in reaction buffer [40 mM Tris (pH 7.5), 20 mM MgCl_2_ and 0.1 mg/ml BSA] at room temperature for 30 min, 4 μM ATP was added and incubated for 30 min at room temperature, then Kinase-Glo™ Reagent was added to detect the remaining amount of ATP, and the results were reflected by luminescence intensity value (RLU). Meanwhile, YycG′ protein with no addition of the derivatives was used as the control and ATP only was used as a blank. The rate of inhibition of kinase phosphorylation (Rp) by the derivatives was calculated by the following equation:

$$\mathrm{Rp}=\frac{\mathrm{RLU}\left(\mathrm{YycG}\hbox{'}+\mathrm{derivatives}+\mathrm{ATP}\right)\hbox{-} \mathrm{RLU}\;\left(\mathrm{YycG}\hbox{'}+\mathrm{ATP}\right)}{\mathrm{RLU}\left(\mathrm{ATP}\;\mathrm{only}\right)\hbox{-} \mathrm{RLU}\;\left(\mathrm{YycG}\hbox{'}+\mathrm{ATP}\right)}\times 100\%$$

IC_50_ (the concentration resulting in 50% inhibition of YycG′ histidine kinase auto-phosphorylation) was obtained by using GraphPad v7.0 software (San Diego, CA, USA). BSA protein without phosphorylation activity were used as the negative control. All experiments were performed in triplicate.

### Growth curves of the planktonic bacteria

After the overnight incubation, liquid cultures of *E. faecium* EF16M64 were sub-cultured (1:200) in fresh TSB medium and H2-60 and H2-81 were doubling diluted (from 1 × MIC to 1/16 × MIC) by TSB medium. After diluted, totally 300 μL dilution were added to growth curve device (Bioscreen C co., Piscataway, USA) in triplicate. The bacterial solution without the derivatives was used as positive control and the effect of the derivatives on bacterial growth was automatically monitored every hour by measuring the optical density at 600 nm (OD_600_) continuously for 16 h. The experiments were performed in triplicate.

### Time-killing assay

Time-killing curve of single dose of two derivatives and combination with vancomycin, linezolid, ampicillin, or daptomycin at concentrations of 4 × MIC respectively were assayed with the suspensions of *E. faecium* EF16M64 (∼1 × 10^6^ CFU/mL). Time-killing experiments were performed at 37 °C with shaking at 220 rpm under aerobic conditions according to previously reported with some modifications [34]. Aliquots (1 mL) were removed at different time points (0, 1, 3, 5, 7, 11, and 24 h), serially diluted with sterile saline, and 100 μL of bacteria were evenly coated on MHA plate and cultured at 37 °C for 24 h, bacterial colonies were counted and determined by plotting log_10_ colony counts (CFU/mL) against time. Bacteria treated with 0.1% DMSO served as a control. The results were independently presented as the mean ± standard deviations (SD) in triplicate.

### Determination of *E. faecium* biofilm formation

Biofilm formation assays were performed by using the methods described in our previous study with minor modification [35]. Briefly, overnight cultures of *E. faecium* EF16M64 were diluted 1:200 with fresh TSBG (TSB plus 0.25% glucose) and inoculated into 96-well polystyrene microtiter plates (200 μL/well, Costar3599, Corning). After incubation at 37 °C for 24 h, the supernatant of the unattached cells was removed and washed with deionized water for three times, adherent biofilms in the plates were fixed with 95% methanol, stained with 1% crystal violet for 20 min and washed, and then dried in air for 2 h. The optical density at 570 nm (OD_570_) was tested by micro plate spectrophotometer (DTX880, Beckman Coulter, USA). *E. faecium* strains EF16M64 and EF16M2 were used as the biofilm-positive and -negative controls, respectively. According to the literature [29], the biofilm formation phenotypes were divided into four categories. The cut-off value of OD_c_ was defined as three standard deviations above the mean OD of the negative control. The classification is as follows: OD_570_ ≤ OD_c_, no biofilm formation, OD_c_<OD_570_ ≤ 2 × OD_c_, weak biofilm formation, 2 × OD_c_<OD_570_ ≤ 4 × OD_c_, medium biofilm formation, OD_570_>4 × OD_c_, strong biofilm formation.

### Anti-biofilm activity of two thiazolidione derivatives with sub-MIC concentration

The inhibitory effect of two derivatives (H2-60 and H2-81) against the biofilms formation of *E. faecium* for 24 h was observed in TSBG medium using a semi-quantitative assay. An overnight bacteria culture was diluted 1:200 into TSBG. Aliquots of the inhibitors at 1/2 × MIC concentrations were mixed with the same volume of the bacterial cultures in TSBG, added to 96-well polystyrene plates in triplicate, and co-cultured under static conditions for 24 h. After washing with sterile saline, the biofilms were stained with crystal violet and the OD_570_ was measured. Wells with no derivatives were used as positive control. All the experiments were repeated in triplicate, and the data represent the means ± standard deviations (SD).

### Determination of cell viability in mature biofilms by confocal laser scanning microscope

The effect of two thiazolidione derivatives on cell viability in mature biofilms (24 h) was determined using the Live/Dead Bacterial Viability method (Live/Dead BacLight, Molecular Probes, USA) with SYTO 9 and propidium iodide (PI) dyes to stain live and dead cells within mature biofilms [36]. Overnight cultures of *E. faecium* EF16M64 was grown in cell-culture dishes (Fluorodish, FD35–100) with TSBG medium and incubated at 37 °C for 24 h. Planktonic bacteria were then removed and discarded, and fresh TSBG containing the derivatives monotherapy (at MIC concentrations) or the derivatives combined with a final concentration of 8 × MIC of ampicillin and daptomycin were added and incubated at 37 °C for a further 72 h, exchanging the media every 24 h for fresh media containing appropriate derivatives. After staining, mature biofilms were observed under a confocal laser scanning microscope (CLSM, Leica) with oil-immersion objective. IMARIS 7.0.0 software (Bitplane) was used to edit and analyze the original images. Green and red fluorescence represented the viable and dead cells, respectively. The PI/total percentage, representing the proportion of dead cells within the mature biofilm, was estimated using ImageJ software (Rawak Software Inc., Stuttgart, Germany). This assay was performed in triplicate and similar results were obtained.

### Statistical analysis

Experiments were performed in triplicate and repeated at least three times. Student’s t-test or nonparametric Mann–Whitney U test was utilized for data comparison. Differences in means were considered significant when *P* < 0.05. All data were analyzed using the statistical software SPSS (version 14.0; SPSS, Chicago, IL, United States).

## Supplementary Information

**Additional file 1 **: **Table S1**. MIC values of YycG inhibitors, ampicillin vancomycin linezolid and daptomycin against *E. faecium* EF16M64. **Table S2**. Sequence analysis of *Enterococcus faecium* DO YycG with other homologues*.* **Table S3**. MICs and MDR phenotypes of two thiazolidione derivatives and clinical relevant agents against 102 clinical *E faecium* strains. **Table S4**. The RLU values of the kinase activities with different concentration of H2-60 and H2-81*.*

## Data Availability

All data generated or analyzed during this study are included in this published article [and its supplementary information files].

## Abbreviations

HK: Histidine kinase
EPS: Extracellular polymeric substances
TCSs: Two-component systems
CLSM: Confocal laser scanning microscope
