# Supplementary material for: In vitro activities of thiazolidione derivatives combined with daptomycin against clinical Enterococcus faecium strains
Source: BMC Microbiol. 2022 Jan 7;22:16. doi: 10.1186/s12866-021-02423-8 (PMC8740470; doi:10.1186/s12866-021-02423-8)
Supplement: Supplementary file 1 — Additional file 1 : Table S1. MIC values of YycG inhibitors, ampicillin vancomycin linezolid and daptomycin against E. faecium EF16M64. Table S2. Sequence analysis of Enterococcus faecium DO YycG with other homologues. Table S3. MICs and MDR phenotypes of two thiazolidione derivatives and clinical relevant agents against 102 clinical E faecium strains. Table S4. The RLU values of the kinase activities with different concentration of H2-60 and H2-81. [file 12866_2021_2423_MOESM1_ESM.docx]

**Table S1 MIC values of YycG inhibitors, ampicillin vancomycin linezolid and daptomycin against *E. faecium* EF16M64.**

| Compounds/Antibiotics | Minimal inhibitory concentration (MIC) |
| --- | --- |
| H2-60 | 3.13μM or 1.62 mg/L |
| H2-81 | 3.13μM or 1.57 mg/L |
| Ampicillin | 1mg/L |
| Vancomycin | 1 mg/L |
| Linezolid | 2 mg/L |
| Daptomycin | 1 mg/L |

**Table S2. Sequence analysis of *Enterococcus faecium* DO YycG with other homologues*.***

| **Strain** | **YycG (Num. of aa)** | **Identical (%)** |
| --- | --- | --- |
| *E. faecalis OG1RF* | 611 | 87.6 |
| *S. epidermidis ATCC 12228* | 610 | 59.1 |
| *S. epidermidis str. 1457* | 610 | 59.0 |
| *S. epidermidis RP62A* | 610 | 59.0 |
| *S. aureus str. Mu50* | 608 | 60.1 |
| *S. aureus str. MW2* | 608 | 60.1 |
| *S. aureus str. N315* | 608 | 60.1 |
| *S. aureus str. MSSA476* | 608 | 60.1 |

**Table S3. MICs and MDR phenotypes of** **two thiazolidione derivatives and clinical relevant agents against 102 clinical *E faecium* strains.**

| **No.** | **strains** | **Gender** | **Source** | **MIC value**  **(μM)** | | **MDR Phenotypes** | | | | | | | | | | |
| --- | --- | --- | --- | --- | --- | --- | --- | --- | --- | --- | --- | --- | --- | --- | --- | --- |
|  |  |  |  | **H2-60** | **H2-81** | **LZD** | **VAN** | **AMP** | **DAP** | **TEC** | **TCY** | **CIP** | **ERY** | **GEH** | **NIT** | **RIF** |
| 1 | EF16M1 | F | Urine | 6.25 | 12.5 | S | S | I | SDD | S | R | R | R | R | R | R |
| 2 | EF16M2 | F | Ear swab | 6.25 | 12.5 | S | I | I | R | S | R | R | R | R | R | R |
| 3 | EF16M3 | M | Urine | 6.25 | 12.5 | S | S | I | SDD | S | S | R | R | S | I | R |
| 4 | EF16M5 | F | Urine | 6.25 | 6.25 | S | S | I | S | S | R | R | R | S | I | R |
| 5 | EF16M7 | F | Urine | 6.25 | 6.25 | S | S | I | S | S | R | R | R | R | I | R |
| 6 | EF16M8 | F | Urine | 6.25 | 6.25 | I | S | I | SDD | S | R | R | R | R | I | S |
| 7 | EF16M9 | F | Urine | 6.25 | 6.25 | S | S | I | S | S | R | S | R | R | I | R |
| 8 | EF16M10 | F | Catheter | 6.25 | 12.5 | S | I | I | R | S | I | S | R | R | R | R |
| 9 | EF16M11 | F | Urine | 6.25 | 12.5 | S | S | I | S | S | R | S | R | R | S | R |
| 10 | EF16M12 | F | Urine | 6.25 | 6.25 | S | S | I | S | S | R | S | R | R | S | R |
| 11 | EF16M13 | M | Catheter | 6.25 | 6.25 | S | S | I | SDD | S | R | S | R | R | R | R |
| 12 | EF16M14 | M | Urine | 6.25 | 6.25 | S | S | I | S | S | I | S | R | R | S | R |
| 13 | EF16M15 | F | Urine | 6.25 | 6.25 | S | S | I | SDD | S | R | S | R | R | I | R |
| 14 | EF16M16 | M | Urine | 6.25 | 12.5 | S | S | I | SDD | S | R | S | R | R | R | R |
| 15 | EF16M17 | M | Catheter | 6.25 | 6.25 | S | S | I | SDD | S | R | S | R | R | R | R |
| 16 | EF16M19 | M | Catheter | 6.25 | 6.25 | S | S | I | SDD | S | R | S | R | R | I | R |
| 17 | EF16M20 | M | Catheter | 6.25 | 12.5 | S | S | I | SDD | S | R | S | R | R | S | R |
| 18 | EF16M21 | F | Urine | 6.25 | 12.5 | S | S | I | SDD | S | R | R | R | R | R | S |
| 19 | EF16M22 | F | Urine | 6.25 | 12.5 | S | S | I | SDD | S | R | R | R | R | R | R |
| 20 | EF16M23 | F | Pleural effusion | 6.25 | 12.5 | S | S | I | SDD | S | R | R | R | R | R | S |
| 21 | EF16M24 | M | Catheter | 6.25 | 12.5 | S | S | I | SDD | S | R | S | R | R | I | R |
| 22 | EF16M25 | M | Urine | 6.25 | 12.5 | S | S | I | SDD | S | R | R | R | R | R | R |
| 23 | EF16M27 | F | Secretions | 6.25 | 6.25 | S | S | I | SDD | I | R | I | R | R | I | R |
| 24 | EF16M28 | F | Urine | 6.25 | 12.5 | S | S | I | SDD | S | R | S | R | R | R | R |
| 25 | EF16M29 | M | Urine | 6.25 | 12.5 | S | I | I | R | S | R | R | R | R | R | R |
| 26 | EF16M30 | F | Urine | 6.25 | 12.5 | S | S | I | SDD | S | R | R | R | R | I | R |
| 27 | EF16M31 | F | Urine | 6.25 | 12.5 | S | S | I | SDD | S | R | R | R | R | R | R |
| 28 | EF16M32 | M | Urine | 6.25 | 12.5 | I | S | I | S | S | R | R | R | R | R | R |
| 29 | EF16M33 | F | Secretions | 6.25 | 6.25 | S | S | I | SDD | S | R | R | R | S | I | R |
| 30 | EF16M34 | M | Urine | 6.25 | 12.5 | S | S | I | SDD | S | S | R | R | R | R | R |
| 31 | EF16M35 | M | Urine | 6.25 | 6.25 | S | S | I | SDD | S | R | R | I | R | R | R |
| 32 | EF16M36 | M | Feces | 6.25 | 12.5 | S | S | I | S | S | S | R | R | R | I | R |
| 33 | EF16M37 | F | Feces | 6.25 | 12.5 | S | S | I | S | S | S | R | R | S | R | R |
| 34 | EF16M38 | M | Urine | 6.25 | 12.5 | S | S | I | S | S | S | R | R | S | I | R |
| 35 | EF16M39 | F | Pus | 6.25 | 12.5 | S | S | S | SDD | S | R | I | R | S | R | R |
| 36 | EF16M40 | M | Urine | 6.25 | 6.25 | S | S | I | SDD | S | I | R | R | R | R | R |
| 37 | EF16M41 | F | Urine | 6.25 | 12.5 | S | S | I | S | S | I | S | R | S | I | S |
| 38 | EF16M42 | M | Sputum | 6.25 | 12.5 | S | S | S | SDD | S | S | R | R | S | R | R |
| 39 | EF16M43 | F | Catheter | 6.25 | 12.5 | R | S | I | R | S | S | R | R | R | R | R |
| 40 | EF16M44 | M | Pus | 6.25 | 6.25 | S | S | S | S | S | S | S | R | S | R | S |
| 41 | EF16M45 | M | Blood | 6.25 | 12.5 | S | S | I | S | S | S | S | I | S | R | S |
| 42 | EF16M46 | M | Blood | 6.25 | 6.25 | S | S | I | SDD | S | R | S | R | R | S | R |
| 43 | EF16M47 | F | Urine | 6.25 | 6.25 | S | S | I | S | S | S | S | R | R | R | R |
| 44 | EF16M48 | M | Catheter | 6.25 | 6.25 | S | S | I | S | S | R | S | R | R | S | R |
| 45 | EF16M49 | F | Blood | 6.25 | 6.25 | S | S | I | S | S | R | R | R | R | I | R |
| 46 | EF16M50 | M | Catheter | 6.25 | 6.25 | S | S | I | SDD | S | R | S | R | R | R | R |
| 47 | EF16M51 | M | Urine | 6.25 | 6.25 | S | S | S | SDD | S | R | I | R | S | I | S |
| 48 | EF16M52 | M | Catheter | 6.25 | 6.25 | S | S | I | SDD | S | R | S | R | R | I | R |
| 49 | EF16M53 | F | Urine | 6.25 | 6.25 | S | S | I | SDD | S | R | S | R | R | R | R |
| 50 | EF16M54 | M | Urine | 6.25 | 6.25 | S | S | I | SDD | S | R | R | R | R | R | R |
| 51 | EF16M55 | F | Ear swab | 6.25 | 6.25 | S | S | I | S | S | R | R | R | R | I | R |
| 52 | EF16M56 | M | Blood | 6.25 | 6.25 | S | S | I | S | S | S | R | R | R | R | R |
| 53 | EF16M57 | F | Secretions | 6.25 | 12.5 | S | S | I | SDD | S | R | R | R | R | I | R |
| 54 | EF16M58 | M | Blood | 6.25 | 6.25 | S | S | I | SDD | S | S | R | R | R | R | R |
| 55 | EF16M59 | F | Urine | 6.25 | 6.25 | S | S | I | S | S | I | R | R | S | R | R |
| 56 | EF16M60 | F | Urine | 6.25 | 6.25 | S | S | I | SDD | S | I | R | R | S | I | R |
| 57 | EF16M61 | F | Urine | 6.25 | 6.25 | S | S | I | SDD | S | I | S | S | R | R | R |
| 58 | EF16M62 | F | Urine | 6.25 | 6.25 | S | S | I | SDD | S | I | R | R | S | R | R |
| 59 | EF16M63 | M | Drainage fluid | 6.25 | 6.25 | S | S | I | SDD | S | I | R | R | S | R | R |
| 60 | EF16M64 | F | Pus | 3.13 | 3.13 | S | S | S | S | S | S | S | R | S | I | R |
| 61 | EF16M65 | M | Catheter | 6.25 | 6.25 | S | S | I | SDD | S | R | R | R | S | R | R |
| 62 | EF16M66 | F | Blood | 6.25 | 6.25 | S | S | I | SDD | S | I | R | S | S | R | R |
| 63 | EF16M67 | F | Urine | 6.25 | 3.13 | S | S | I | SDD | S | I | R | R | S | S | R |
| 64 | EF16M68 | M | Blood | 6.25 | 6.25 | S | S | I | S | S | I | R | R | S | R | R |
| 65 | NEFM1 | F | Urine | 25 | 25 | S | S | I | S | S | S | R | R | R | R | R |
| 66 | NEFM2 | F | Secretions | 25 | 25 | S | S | I | S | S | R | R | R | R | I | R |
| 67 | NEFM4 | M | Bronchoalveolar lavage fluid | 25 | 25 | S | S | I | S | S | R | R | R | R | R | R |
| 68 | NEFM5 | M | Bile | 25 | 25 | S | S | I | S | S | S | R | R | S | R | R |
| 69 | NEFM6 | M | Blood | 12.5 | 12.5 | S | S | I | S | S | R | R | R | R | R | R |
| 70 | NEFM7 | F | Urine | 12.5 | 12.5 | S | S | S | S | S | I | I | I | S | S | I |
| 71 | NEFM8 | F | Bile | 12.5 | 12.5 | I | S | I | S | S | S | S | R | S | R | R |
| 72 | NEFM9 | F | Urine | 12.5 | 12.5 | S | S | I | S | S | S | R | R | R | R | R |
| 73 | NEFM10 | M | Catheter | 12.5 | 12.5 | S | S | I | S | S | R | R | R | S | I | R |
| 74 | NEFM11 | F | Drainage fluid | 12.5 | 12.5 | S | S | I | S | S | S | R | R | R | R | R |
| 75 | NEFM12 | F | Urine | 12.5 | 12.5 | S | S | I | S | S | S | R | R | R | R | S |
| 76 | NEFM13 | M | Secretions | 12.5 | 12.5 | S | S | I | S | S | S | R | R | R | R | S |
| 77 | NEFM14 | M | Urine | 12.5 | 12.5 | S | S | I | S | S | S | R | R | S | R | R |
| 78 | NEFM15 | F | Urine | 12.5 | 12.5 | S | S | I | S | S | S | R | R | R | R | R |
| 79 | NEFM16 | F | Urine | 12.5 | 12.5 | S | S | I | S | S | R | R | S | R | R | R |
| 80 | NEFM17 | F | Urine | 12.5 | 25 | S | S | I | S | S | S | R | R | R | R | R |
| 81 | NEFM18 | F | Bile | 12.5 | 12.5 | S | S | I | S | S | S | R | R | R | R | R |
| 82 | NEFM19 | F | Bile | 12.5 | 12.5 | S | S | I | S | S | S | S | R | S | R | S |
| 83 | NEFM20 | M | Secretions | 6.25 | 6.25 | S | S | I | S | S | S | R | R | R | R | R |
| 84 | NEFM21 | F | Bile | 6.25 | 6.25 | S | S | I | S | S | S | R | R | R | R | R |
| 85 | NEFM22 | F | Urine | 6.25 | 6.25 | S | S | I | S | S | R | R | R | R | I | R |
| 86 | NEFM23 | M | Secretions | 6.25 | 12.5 | S | S | I | SDD | S | I | R | R | R | I | S |
| 87 | NEFM24 | F | Urine | 6.25 | 6.25 | S | S | S | S | S | I | S | S | S | S | S |
| 88 | NEFM25 | F | Urine | 6.25 | 6.25 | S | S | S | S | S | I | S | S | S | S | S |
| 89 | NEFM26 | F | Urine | 6.25 | 6.25 | S | S | I | S | S | S | R | R | R | R | R |
| 90 | NEFM28 | F | Ascites fluid | 6.25 | 6.25 | S | S | I | S | S | S | R | R | S | R | R |
| 91 | NEFM29 | M | Urine | 6.25 | 12.5 | S | S | I | S | S | S | R | R | R | R | R |
| 92 | NEFM30 | F | Bile | 6.25 | 12.5 | S | S | I | S | S | S | I | I | S | I | I |
| 93 | NEFM31 | F | Urine | 6.25 | 6.25 | S | S | I | S | S | S | R | R | S | R | R |
| 94 | NEFM32 | M | Urine | 6.25 | 12.5 | S | S | I | S | S | R | S | R | R | S | R |
| 95 | NEFM34 | F | Urine | 12.5 | 12.5 | S | S | I | S | S | S | R | R | S | R | R |
| 96 | NEFM35 | F | Urine | 12.5 | 12.5 | S | S | S | S | S | I | S | S | S | S | S |
| 97 | NEFM36 | M | Urine | 6.25 | 6.25 | S | S | I | S | S | S | I | S | S | I | I |
| 98 | HAFM12 | M | Blood | 6.25 | 6.25 | S | S | R | S | S | S | S | R | R | S | S |
| 99 | HAFM24 | M | Urine | 12.5 | 12.5 | S | S | R | S | S | S | R | S | R | S | S |
| 100 | HAFM62 | F | Urine | 3.13 | 6.25 | R | I | R | R | S | S | R | S | R | R | S |
| 101 | HAFM75 | M | Urine | 6.25 | 6.25 | S | S | R | S | S | R | R | S | R | I | S |
| 102 | HAFM77 | F | Urine | 6.25 | 6.25 | S | S | R | S | S | R | R | S | R | S | S |

Note: MIC of two thiazolidione derivatives and clinical relevant agents were determined by broth microdilution method according to CLSI guidelines (CLSI-M100-S27). LZD: Linezolid (Oxazolidinones); VAN: Vancomycin (Glycopeptides); AMP: Ampicillin (Penicillins); DAP: Daptomycin (Lipopeptides); TEC: Teicoplanin (Glycopeptides); TCY: Tetracycline (Tetracyclines); CIP: Ciprofloxacin (Fluoroquinolones); ERY: Erythromycin (Macrolides); GEH: high-level gentamicin (Aminoglycosides); NIT: Nitrofurantoin (Nitrofurantoins); RIF: Rifampin (Ansamycins). R: Resistant; I: Intermediate; S: Susceptible; SDD: Susceptible-dose dependent.

**Table S4. The RLU values of the kinase activities with different concentration of H2-60 and H2-81*.***

| **Concentrations**  **(μM)** | | **RLU values of derivatives** | | |
| --- | --- | --- | --- | --- |
|  |  | **H2-60^a^** | **H2-81^a^** | **H2-60^b^** |
| Protein+compounds+ATP group | 200 | 249203 | 248273 | 241838 |
|  | 100 | 201887 | 199674 | 226046 |
|  | 50 | 188635 | 184341 | 218098 |
|  | 25 | 179568 | 178358 | 214389 |
|  | 12.5 | 173776 | 171720 | 209312 |
|  | 6.25 | 171456 | 167813 | 207365 |
|  | 3.13 | 162939 | 163033 | 200512 |
|  | 1.56 | 161641 | 155503 | 197818 |
| Protein'+ATP group | | 176263 | 175312 | 220503 |
| ATP only group | | 184627 | 183692 | 231164 |

^a^ Protein used in H2-60 and H2-81 group were the YycG recombinant protein.

^b^ Protein used in H2-60 group was the BSA protein.
